# Development and Characterization of a Topically Deliverable Prophylactic Against Oxidative Damage in Cochlear Cells

**DOI:** 10.3389/fphar.2022.907516

**Published:** 2022-06-09

**Authors:** Elizabeth M. Arrigali, Monica A. Serban

**Affiliations:** ^1^ Pharmaceutical Sciences and Drug Design Program, Department of Biomedical and Pharmaceutical Sciences, University of Montana, Missoula, MT, United States; ^2^ Montana Biotechnology Center, University of Montana, Missoula, MT, United States

**Keywords:** cochlear cells, prophylactic, oxidative stress, hyaluronan, antioxidant

## Abstract

Hearing loss affects roughly 466 million people worldwide. While the causes of hearing loss are diverse, mechanistically, inflammation and oxidative stress have been identified as major players in hearing loss regardless of pathogenesis. Treatment options remain extremely limited and there is currently no FDA approved drug therapy. Studies indicate that antioxidants such as d-Methionine have shown some protective effects; however, these studies involved systemic or invasive localized delivery methods and highlighted the need for the development of minimally invasive localized therapeutic approaches. Described herein is the development of an antioxidant-conjugated system that shows prophylactic potential against oxidative damage and appears suitable for topical delivery. Specifically, our covalent conjugate of hyaluronan with d-Methionine shows cytocompatibility and protection from oxidative stress in two mouse cochlear cell lines (HEI-OC1 and SV-k1). Mechanistically, the data indicate that the protective effects of the conjugate are due to the hyaluronan-mediated cellular internalization of the antioxidant. Most notably, the conjugate can efficiently permeate through an *in vitro* round window membrane model without the loss of the attached antioxidant, for subsequent delivery of the therapeutic cargo to the hearing sensory cells. Collectively these findings show that the novel conjugate could be a potential topical preventive agent against hearing loss.

## GRAPHICAL ABSTRACT

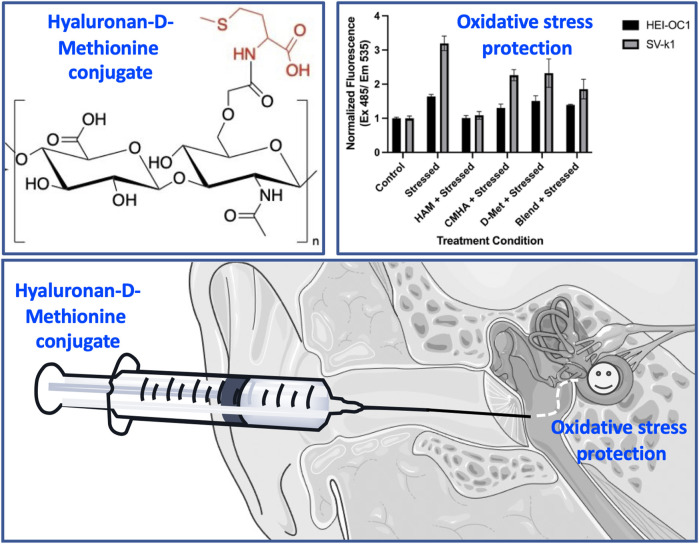

## Background

Worldwide, hearing loss is reportedly affecting more than half a billion people and is the fourth leading contributor to living with a disability; the costs associated with it are greater than $750 billion each year (Brown et al., 2018). Hearing loss can occur for a variety of reasons and can be age related, noise-induced, drug-induced, or caused by viral, bacterial, or chemical agents. Nevertheless, currently there are no Food and Drug Administration (FDA)-approved drugs to prevent it (Ingersoll et al., 2020). Mechanistically, inflammation and oxidative stress have been identified as major players in this pathology regardless of its etiology (Kamogashira et al., 2015; Kurabi et al., 2017).

Animal studies highlighted the protective role of antioxidants against noise-, virus-, age- or drug-induced hearing loss (Le Prell et al., 2007; Tavanai and Mohammadkhani, 2017; Pecha et al., 2020). In noise-induced hearing loss, one study underlined the mitochondrial generation of reactive oxygens species (ROS) and showed that d-Methionine (D-Met) treatment protected animals from temporary threshold shifts (Cheng et al., 2008). Mechanistically, noise-initiated oxidative stress was shown to occur through the release of calcium ions from the endoplasmic reticulum or potential entry from extracellular fluid leading to the release of ROS from mitochondria, and *via* activation of nicotinamide adenine dinucleotide phosphate (NADPH) oxidases (Kurabi et al., 2017). A separate study revealed that excessive ROS is associated with cytomegalovirus (CMV)-induced hearing loss, and intraperitoneal treatment of hearing-impaired mice with D-Met and ACE-Mg (combination of vitamins A, C, E and magnesium) attenuated the extent of hearing loss (Pecha et al., 2020). In presbycusis or age-related hearing loss supplementation with antioxidants was shown to reduce the progression of the disease (Fujimoto and Yamasoba, 2014). Aging, according to the free radical or oxidative stress theory, is considered to be the result of increased oxidative damage (Lin and Flint Beal, 2003). Drug-induced ototoxicity was also linked to oxidative stress. Cisplatin and aminoglycoside antibiotics were shown to drastically increase cellular ROS production (Ravi et al., 1995; Sha and Schacht, 1999).

While animal studies targeting hearing loss *via* oxidative stress reduction reported successful outcomes, human data lag (Kurabi et al., 2017; Pak et al., 2020). Interestingly, while hereditary deafness was reported to benefit from dietary antioxidant supplementation (Green et al., 2016), other studies have shown that antioxidant-enriched diets did not delay the progression of age-related hearing loss (Cheng et al., 2008). We believe that, in contrast to orally administered antioxidants, their topical delivery would result in increased cochlear bioavailability and enhanced therapeutic outcomes. Therefore, our approach for this study was to develop a topically deliverable antioxidant system that could prevent or treat oxidative damage typically associated with hearing loss. Our rationale was to employ an antioxidant ‘carrier’ to permeate ear-specific biological membranes (such as the tympanic membrane or the round window membrane) with the drug cargo and deliver it to the cochlear structures typically affected by oxidative stress.

For this, hyaluronic acid (HA) was selected as the carrier, as this macromolecule has been previously reported to aid in the delivery of drugs into cochlear structures (Chaulagain et al., 2018; Altman et al., 2019; Yu et al., 2021). Specifically, one study reported that pre-treatment of the round window membrane with HA resulted in increased cochlear gene delivery in a guinea pig model (Shibata et al., 2012). A separate clinical study reported that patients treated with a mix of dexamethasone/HA led to better therapeutic outcomes for sudden sensorineural hearing loss compared to patients treated with only dexamethasone (Rogha and Kalkoo, 2017). Moreover, HA has been widely explored as a transdermal drug delivery enhancer, and shown to increase drug permeation through normal skin, seemingly by a combination of cotransport, increased tissue hydration, and modifications of the stratum corneum properties (Witting et al., 2015). HA is a naturally occurring, high-molecular weight polymer consisting of repeating disaccharide units, ubiquitous in the mammalian extracellular matrix (Gupta et al., 2019). Its chemical structure readily lends itself to well characterized chemical modifications and generation of highly biocompatible derivatives (Serban and Skardal, 2019). d-Methionine (D-Met) was selected as the antioxidant as its protective effects against cochlear oxidative stress are well documented (Cheng et al., 2008; Wang et al., 2019; Pecha et al., 2020; Campbell et al., 2021) Additionally, its structure contains a primary amine group suitable for carbodiimide mediated conjugation to carboxyl groups present on the HA backbone (Serban and Skardal, 2019). Presented herein are the syntheses and characterization of hyaluronan-d-Methionine conjugates (HAM) and their effects on cochlear cells exposed to oxidative stress.

## Materials and Methods

### Reagents and Instruments

The following reagents and consumables have been used for this study: hyaluronic acid (HA) MW 850 kDa (Novozymes, Denmark), sodium hydroxide (NaOH, VWR, Radnor, PA), iodoacetic acid (ThermoScientific, Waltham, MA), isopropanol (VWR, Radnor, PA), Whatman filter paper (Fisher, Waltham, MA), hydrochloric acid 6N (HCl, Fisher, Waltham, MA), Slide-A-Lyzer Dialysis Cassettes, 3500 MWCO (ThermoScientific, Waltham, MA), deuterated water (D_2_O, ThermoScientific, Waltham, MA), d-Methionine (D-Met, Waltham, MA), 2-(N-morpholino)ethanesulfonic acid buffer (MES, ThermoScientific, Waltham, MA), 1-ethyl-3-(3- dimethylaminopropyl) carbodiimide (EDC, ThermoScientific, Waltham, MA), lithium citrate buffer (FUJIFILM Wako, Osaka, Japan), Agilent PL Aquagel-OH Mixed H larger molecular weight SEC column (Agilent Technologies, Santa Clara, CA), Agilent PL Aquagel-OH SEC column (Agilent Technologies, Santa Clara, CA).

The following cell lines, cell culture reagents and assay kits have been used for this study: House Ear Institute-Organ of Corti (HEI-OC1) and Stria Vascularis k1 (SV-k1) (Kalinec Lab, UCLA, Los Angeles, CA), three-dimensional (3D) *in vitro* tissue models, Epi-Airway day 7 (MatTek, Ashland, MA), fetal bovine serum (FBS, Corning, Corning, NY), Dulbecco’s Modified Eagle Medium (DMEM, Corning, Corning, NY), Cell-Titer 96 Aqueous One Solution Cell Proliferation Assay (Promega, Madison, WI), CyQUANT NF Cell Proliferation Assay Kit (Molecular Probes, Eugene, OR), phosphate buffered-saline (PBS, Corning, Corning, NY), hydrogen peroxide (H_2_O_2_, Millipore Sigma, St. Louis, MO), chloromethyl derivative of 2′,7′-dichlorodihydrofluorescein diacetate (CM-H2DCFDA, Invitrogen, Waltham, MA), CellEvent Caspase-3/7 Green Detection Reagent (ThermoFisher, Waltham, MA), Trypsin-ethylenediaminetetraacetic acid (trypsin-EDTA, Corning, Corning, NY), NADP/NADPH Assay Kit (Abcam, Waltham, MA), 10 kDa column (Biovision, Milpitas, CA), fluorometric methionine assay kit (Biovision, CA), Ibidi μ -Slide 8 well (Ibidi, Fitchburg, WI), boron-dipyrromethene-labeled HA (HA-BODIPY, Echelon Biosciences, Salt Lake City, UT), MitoTracker Red (Invitrogen, Waltham, MA), LysoTracker Red (Invitrogen, Waltham, MA), bovine serum albumin (BSA, Corning, Corning, NY), formaldehyde solution (VWR, Radnor, PA), 4′,6-diamidino-2-phenylindole nuclear stain (DAPI, ThermoFisher, Waltham, MA).

The following instruments have been used for this study: Varian VNMRS 500 MHz nuclear magnetic resonance spectrometer (Agilent Technologies, Palo Alto, CA), High Speed Amino Acid Analyzer L-8900 (Hitachi, Tokyo, Japan), FilterMax F5 multimode microplate reader (Molecular Devices, San Jose, CA), Leica Stellaris 5 Confocal Microscope (Leica, Germany), Agilent High Pressure Liquid Chromatography instrument with a UV-VIS detector (Agilent, Santa Clara, CA) additionally equipped with an OptiLab differential refractive index (RI) detector (Wyatt Technologies, Santa Barbara, CA), and a miniDawn 3-angle/multi-angle light scattering (MALS) detector (Wyatt Technologies, Santa Barbara, CA).

### Synthesis of Carboxymethylated Hyaluronic Acid (CMHA)

HA was derivatized with carboxymethyl functionalities to increase the conjugation sites available for the covalent attachment of molecules containing primary amines. Briefly, 2 g of HA, MW 850 kDa were added to 20 ml of 45% w/v NaOH and allowed to activate at room temperature (RT) for 2 h. In parallel, 2 g of iodoacetic acid were dissolved in 50 ml of isopropanol. The viscous HA solution was added to 150 ml of isopropanol, then the iodoacetic acid solution was added to the activated HA/isopropanol solution. The reaction mix was allowed to react for 2 h and was then filtered using a Buchner funnel with a #2 Whatman filter paper. The white filter cake obtained after filtration was dissolved in 200 ml of deionized water (diH_2_O) and the pH of the resulting solution was neutralized using 6N HCl. The resulting CMHA solution was then loaded into 3500 MWCO Slide-A-Lyzer Dialysis Cassettes and dialyzed for 72 h with a minimum of five water changes per 24 h to remove residual reagent and salts. After dialysis the CMHA solution was removed from the cassettes, frozen by placement in a −80°C freezer for a minimum of 4 h and subsequently lyophilized. The reaction yielded 1.580 g of CMHA. The carboxymethylation was confirmed by proton nuclear magnetic resonance (^1^H-NMR).

### Synthesis of CMHA-d-Methionine (HAM) Conjugates

D-Met was chemically conjugated to CMHA using carbodiimide chemistry. Briefly, 1 g of CMHA was dissolved into 200 ml of MES buffer in a 450 ml beaker covered with parafilm. The solution was stirred for approximately 25 min until CMHA was fully dissolved. Next, 4.4 g of D-Met were added to the solution and allowed to dissolve (∼5 min). For the covalent conjugation of the antioxidant to CMHA, 2 g of EDC, a zero-length crosslinker, was added to the solution and allowed to react at RT for 20 h. Subsequently, the reaction was neutralized to a pH of 7.0 with an NaOH solution. The reaction mix was then transferred to 3500 MWCO Slide-A-Lyzer Dialysis Cassettes and dialyzed for 72 h with a minimum of five water changes per 24 h to remove residual D-Met. After dialysis the HAM solution was removed from the cassettes, frozen by placement in a −80 °C freezer for a minimum of 4 h and subsequently lyophilized. The reaction yielded 1.214 g of HAM. The conjugation was confirmed by ^1^H-NMR.

### ^1^H-NMR Analysis of Compounds

To confirm the structures and purity of CMHA and HAM, 1H-NMR spectral data were obtained using a Varian VNMRS 500 MHz at 20°C. For the analyses, samples were dissolved in deuterium oxide at a concentration of 5 mg/ml and all spectra were referenced to the residual solvent peak (H2O) at δ= 4.65 ppm .

### Colorimetric Cell Viability/Proliferation Assay

HEI-OC1 and SV-k1 were seeded in a 96-well plate with a seeding density of 1.5 × 10^5^ cells/mL in 100μL of DMEM media with 2% FBS and incubated at 33°C/10% CO_2_ overnight (Kalinec et al., 2016). Subsequently the media was aspirated, replaced with sterile filtered media containing HAM conjugate and controls (CMHA only, D-Met only and CMHA/D-Met blend, all at concentrations equivalent to HAM) and plates were incubated overnight. The viability and proliferation of the cells was quantified with a Cell-Titer 96 Aqueous One Solution Cell Proliferation Assay and the absorbance at 450 nm was measured with a FilterMax F5 multimode microplate reader.

### Fluorescent Cell Viability/Proliferation Assay

HEI-OC1 and SV-k1 cells were used to assess the proliferation of cells treated with the HAM conjugate and controls. Cells were seeded in a 96-well plate with a seeding density of 1.5 × 10^5^ cells/mL in 100 μL of DMEM media with 2% FBS and incubated at 33°C/10% CO_2_. The cells were allowed to adhere overnight, and the media was aspirated and replaced with sterile filtered media containing HAM conjugate and controls then incubated overnight. The proliferation of cells was quantified with a CyQUANT NF Cell Proliferation Assay Kit and the florescence (ex/em 485/530 nm) measured using the FilterMax F5 multimode microplate reader.

### Reactive Oxygen Species (ROS) Detection

HEI-OC1 and SV-k1 cells, respectively, were seeded in a 96-well plate at a seeding density of 2 × 10^5^ cells/mL in DMEM media with 10% FBS and incubated at 33°C/10% CO_2_. The cells were allowed to adhere 24 h, then the media was aspirated and replaced with sterile filtered media containing HAM conjugates and controls, followed by incubation for 24 h. The media was then aspirated and replaced with 1 μM CM-H2DCFDA in PBS and incubated for and incubated for 30 min. The experimental conditions used for the ROS assays associated with this project were experimentally determined by our group to yield appropriate stress responses without causing cell detachment from the culture plates. The reagent was then aspirated and replaced with either PBS for control or 0.5 mM H_2_O_2_ in PBS and incubated for 30 min. Media was then aspirated, and wells were washed 2 times with PBS. The amount of ROS was quantified using florescence (ex/em 495/530 nm) with the FilterMax F5 multimode microplate reader.

### Detection of Apoptosis Markers

HEI-OC1 and SV-k1, respectively, were seeded in a 96-well plate with a seeding density of 2 × 10^5^ cells/mL in DMEM media with 10% FBS and incubated at 33°C/10% CO_2_. The cells were allowed to adhere 24 h, then the media was aspirated and replaced with sterile filtered media containing HAM conjugate and controls and then incubated for 24 h. The media was then aspirated and replaced with either PBS for control or 0.5 mM H_2_O_2_ in PBS and incubated for 30 min. Media was then aspirated, and wells were washed 2 times with PBS. DMEM with 10% FBS was added to each well and the plate was incubated for 48 h at 33°C/10% CO_2_. The amount of apoptosis was assessed with 4μM CellEvent Caspase-3/7 Green Detection Reagent and the florescence (ex/em 495/530 nm) was read using the FilterMax F5 multimode microplate reader.

### Nicotinamide Adenine Dinucleotide Phosphate (NADPH/NADP) Quantification

HEI-OC1 and SV-k1, respectively, were seeded in a 75 cm^2^ flask with a seeding density of 1 × 10^6^ cells/mL in DMEM media with 10% FBS and incubated at 33°C/10% CO_2_ for 48 h. Media was then aspirated and replaced with sterile filtered media containing HAM conjugate and controls and incubated for 24 h. Treatment solutions were aspirated, and flasks were washed with 1x PBS two times. Cells were then stressed with fresh 0.5 mM H_2_O_2_ or PBS only for controls for 30 min. Stressing solution was then aspirated and flasks were washed two times with 1x PBS. Cells were detached using 0.25% trypsin-EDTA. Cells and and trypsin solution was centrifuged at 300 RCF for 5 min and cells were counted using Countess Cell Counter. Cell numbers for each group were normalized to 4 × 10^6^ cells. Cells were washed two times with 1x PBS to remove any remaining trypsin then processed according to the NADP/NADPH Assay Kit manufacture’s protocol for cellular lysates. Sample quantification was performed *via* absorbance detection at 450 nm with a microplate reader.

### Methionine Determination

A fluorometric methionine assay kit was used for this. HEI-OC1 and SV-k1, respectively, were seeded in a 12 well plate with 0.5 ml of cell solution seeded at 1 × 10^6^ cells/mL in DMEM media with 10% FBS and incubated at 33°C/10% CO_2_ for 48 h. Media was then aspirated and replaced with sterile filtered media containing HAM conjugate and controls and incubated for 24 h. Cells were detached with 0.25% trypsin-EDTA and pelleted *via* centrifugation at 300 RCF. The cell pellets were resuspended in 1x PBS two times to remove any traces of trypsin. Cells were then counted with Countess Cell Counter, and each treatment group was normalized to 2 × 10^6^ cells. Subsequently, 500 μL of assay kit buffer were added to each treatment group and vortexed. The solutions were then centrifuged at 17,000 RCF for 15 min. The supernatants were transferred to 10 kDa column and centrifuged for 10 min at 10,000 RCF. The ultrafiltrates were collected and further processed per the manufacturer’s protocol using cell lysates. Samples were analyzed *via* detection at fluorescence (ex/em 535/595 nm) on a microplate reader.

### HA Internalization Analyses

HEI-OC1 and SV-k1, respectively, were plated at 5 × 10^4^ cells/mL with 200 μL into each well of an Ibidi μ -Slide 8 well. Cells were incubated for 48 h at 33°C/10% CO_2_. Media was aspirated and 200 μL of 50 μM HA BODIPY, 50 nM MitoTracker Red, 50 nM LysoTracker Red and 1x PBS with 0.1% BSA were added to respective wells for 2.5 h and incubated at 33°C/10% CO_2._ Following incubation, organelle stains and HA were aspirated, and wells were washed two times with 1x PBS with 0.1% BSA. Cells were then fixed with 10% formaldehyde solution for 1 h and incubated at RT. The formaldehyde was then removed, and wells were washed two times with 1x PBS and 0.1% BSA, followed by the addition of 1μM DAPI solution to each well and incubation at RT for 5 min. Subsequently, DAPI was removed, wells were washed two times with 1x PBS, and 200μL of 1x PBS were added to every well for imaging with a Leica Stellaris 5 confocal microscope, by using the ×40 objective.

### Round Window Membrane Model Permeation Studies

An Epi-Airway Day 7 3D *in vitro* tissue model was used as mimetic of the round window membrane (Rask-Andersen et al., 2015; Liebesny et al., 2018). HAM conjugate solution at 50 mg/ml and individual controls diluted in 1x PBS, respectively, were added to the top of Epi-Airway Day 7 3D tissues in transwell plates, with 500 μL of 1x PBS in the receiver compartment of a 12 well plate. The plate and tissues were incubated at 37 °C/5% CO_2_ for 24 h. After 24 h the receiver solution was collected and filtered with a 0.1 μm filter prior to size exclusion chromatography (SEC) analyses.

### SEC Analyses

The filtered samples were immediately frozen until ready to use. For analyses, 50 μL of filtered sample were run through an Agilent PL Aquagel-OH Mixed H SEC column for higher molecular weights (detection range: 6 kDa - 10 MDa) for HAM or CMHA detection and analysis, and collections of each sample run were conducted between 8 and 11 min of the 22-min total run time. These collections were then frozen until later use. After all samples were run through the SEC column for larger MW, 50 μL of each collection were run through an Agilent PL Aquagel-OH SEC column for lower molecular weights (detection range: 100 Da–20 kDa) to detect for D-Met (MW = 149 Da). The concentrations of HAM and CMHA, respectively, that permeated through the tissue models of the larger MW SEC column where quantified with the RI detector. Additionally, tissue permeants were also analyzed with the UV-VIS detector which specifically detects D-Met at 235 nm. Standard curves of HAM, CMHA and D-Met, respectively, were created *via* area under the curve at 235 nm.

### Statistical Analyses

All experiments were run in triplicates or greater and all data were presented as mean ± standard deviation of the mean. Student t-tests were performed for 2-group comparisons while one-way ANOVA with multiple comparisons were performed for more than 2-group comparisons with confidence limits of 95% considered significant.

## Results

### Synthesis and Characterization of HAM Conjugates

**FIGURE 1:**
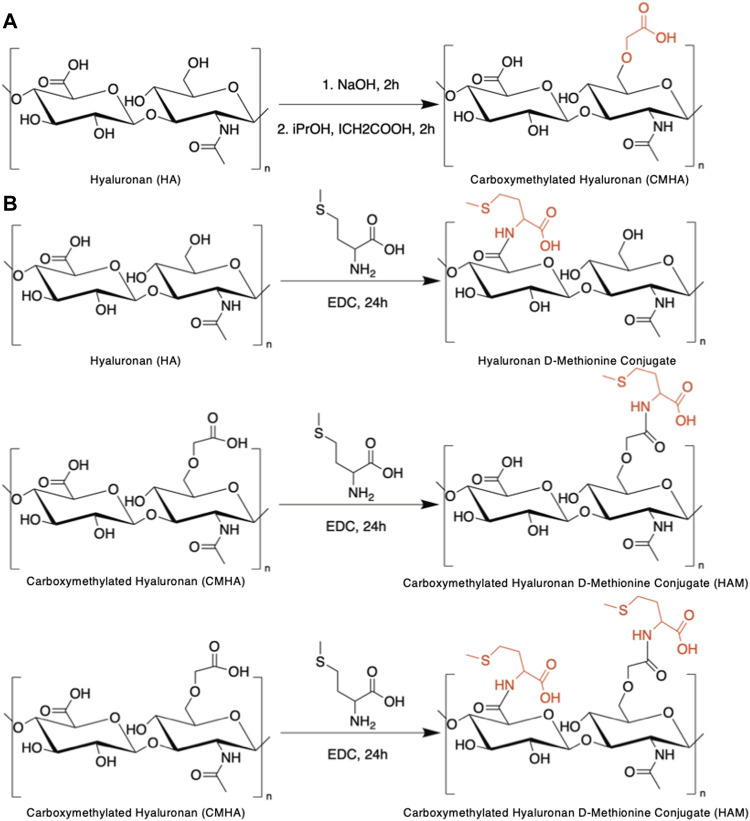
Syntheses of HAM conjugate. **(A)** Reaction scheme for the chemical functionalization of hyaluronic acid (HA) to yield carboxymethylated hyaluronic acid (CMHA). **(B)** Conjugation of d-Methionine to CMHA to yield the hyaluronan-d-Methionine conjugate (HAM).

For the conjugate syntheses, D-Met was selected as the antioxidant of choice based on its reported cochlea-specific antioxidant protection and chemical structure. Considering HA’s chemical structure (Figure 1A), our strategy for chemical conjugation was to use carbodiimide chemistry to create a covalent bond between the carboxyl functionalities existing on the HA backbone and the primary amine groups of D-Met. This synthetic scheme is well characterized, uses a zero-length crosslinker that directly couples the two compounds of interest without adding any additional bulk, and requires mild reaction conditions. The HA backbone, that consists of repeating, identical disaccharide units, intrinsically contains one carboxyl functionality per repeat (Figure 1A). To increase the amount of D-Met that could be covalently attached to the HA backbone, in a first step the HA backbone was enriched in carboxyl functionalities. This was achieved *via* the addition of carboxymethyl groups to the primary hydroxyl functionalities of the disaccharide units to yield CMHA. The successful structural change was confirmed by ^1^H-NMR (Supplementary Figure S1), and based on the spectra, the extent of the functionalization was assessed to be approximately 58% (Supplementary Figure S2). The CMHA was then used as a substrate for D-Met attachment according to the reaction scheme illustrated in Figure 1B. Three different HA to D-Met stoichiometries were used (2X, 10X, 20X molar excess of D-Met to HA disaccharide unit), and the amount of conjugated D-Met was quantified by amino acid analysis.

Our data (Supplementary Table S1) indicated that the amount of conjugated D-Met was higher for the 10X reaction stoichiometry compared to the 2X and was comparable with the 20X reaction mix (within the detection method variability range). We therefore conducted all our subsequent experimental data with the 10X reaction product, which was determined to contain ∼427 nmol/mg of D-Met. As controls, 10X HAM equivalent amounts of CMHA and D-Met were used either alone or as blend (Supplementary Table S2).

### HAM Cytocompatibility

**FIGURE 2:**
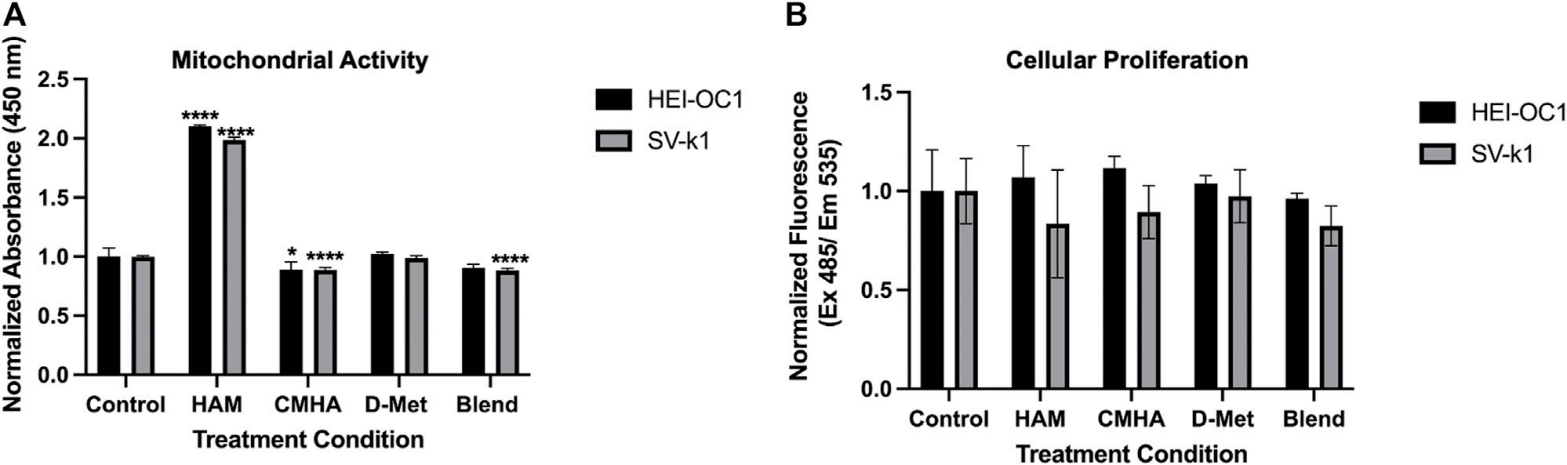
Cytocompatibility of the HAM conjugate. **(A)** MTS assay data indicating that when HAM is added to cells it impacts the activity of mitochondrial enzymes. Data presented are mean ± SD. One-way ANOVA with Tukey’s multiple comparisons run for each cell type individually. HEI-OC1 ANOVA results compared to the control: HAM *p* < 0.0001 for significant increase, CMHA *p* = 0.0305, D-Met *p* = 0.9607, and blend *p* = 0.0623. SV-k1 ANOVA results compared to the control: HAM *p* < 0.0001 for significant increase, CMHA *p* < 0.0001 for significant decrease, D-Met *p* = 0.9586, and blend *p* < 0.0001 for significant decrease. ****p < 0.0001, *p < 0.05. **(B)** CyQuant Cellular Proliferation assay data indicating that HAM addition to the cells does not induce cell proliferation. Data presented are mean ± SD. One-way ANOVA with Tukey’s multiple comparisons run for each cell type individually. HEI-OC1 ANOVA results compared to the control: HAM *p* = 0.9288, CMHA *p* = 0.6659, D-Met *p* = 0.9919 and blend *p* = 0.9915. SV-k1 ANOVA results compared to the control: HAM *p* = 0.6557, CMHA *p* = 0.8984, D-Met *p* = 0.9995, and blend *p* = 0.6025.

To evaluate the potential applicability of HAM as a prophylactic or therapeutic against cochlear oxidative cell damage, typically associated with hearing loss, HEI-OC1 and SV-k1 cochlear cell lines were treated with HAM, CMHA, D-Met and CMHA + D-Met blend, respectively, and assayed for cell viability. Two different assays were used for this. The first one was a colorimetric methyl tetrazolium salt (MTS)-based assay that quantifies cell viability as a function of the cellular enzymatic reduction of the MTS which results in the development of a red color in the assay solution. The intensity of the color typically directly correlates with the number of viable cells. The second assay used was a more sensitive quantification method that relies on the binding of a cell-permeant fluorescent dye to the nucleic acid of live cells. The colorimetric assay results (Figure 2A) appear to indicate increased cell numbers/proliferation in cells treated with HAM, compared to those treated with CMHA, D-Met and CMHA + D-Met, or control, media-treated ones. Specifically, compared to the control group, the number of HAM treated cells appeared significantly higher, while CMHA and CMHA + D-Met, treated cells exhibited slightly lower viability. Only D-Met treatment did not seem to affect cell viability/number compared to the control. The colorimetric assay, however, is based on the enzymatical reduction of the assay reagent, particularly, it reflects the activity of mitochondrial enzymes. A validation of the MTS assay-observed trends was expected through the CyQuant assay. Interestingly, the fluorescent CyQuant assay results appear to indicate no statistically significant differences in cell numbers/viability for any of the treatment groups compared to the control, media-treated ones (Figure 2B). Cumulatively, these results seem to indicate that HAM is cytocompatible, and when added to cells it impacts the activity of mitochondrial enzymes but does not induce cell proliferation in HEI-OC1 or SV-k1 cells.

### HAM Protective Effects in Oxidatively Stressed Cochlear Cells

**FIGURE 3:**
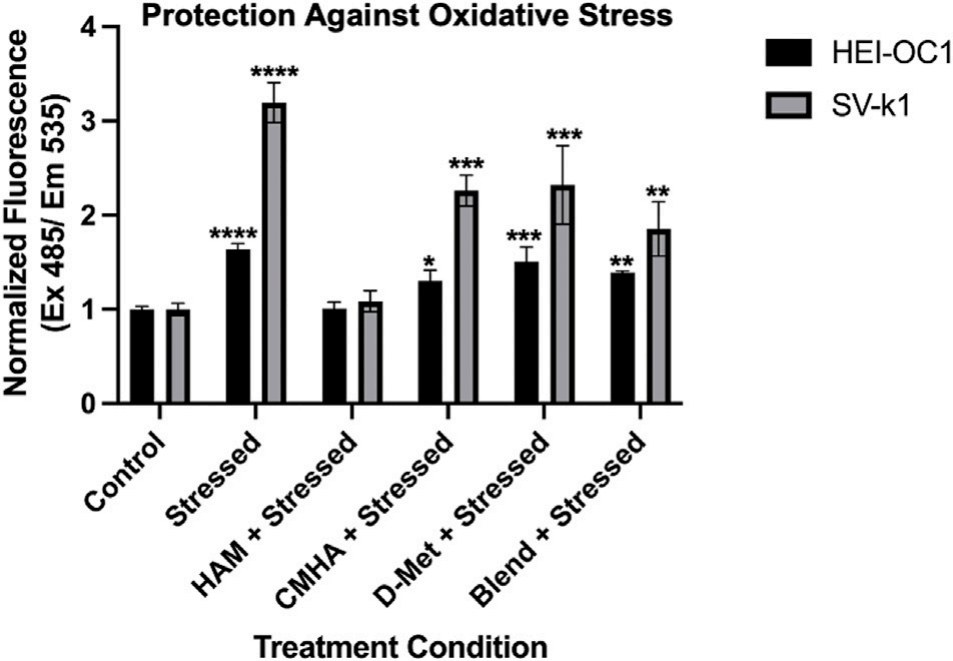
Oxidative stress protective effects of the conjugate in cochlear cell lines. Data presented are mean ± SD. One-way ANOVA with Tukey’s multiple comparisons run for each cell type individually. HEI-OC1 ANOVA results compared to unstressed untreated control: stressed *p* < 0.0001, HAM *p* > 0.9999, CMHA *p* = 0.0118, D-Met *p* = 0.0001, and blend *p* = 0.0016. ****p < 0.0001, ***p = 0.0001, **p = 0.01, and *p = 0.05. SV-k1 ANOVA compared to control: Stressed *p* < 0.0001, HAM *p* = 0.9974, CMHA *p* = 0.0004, D-Met *p* = 0.0002, and blend *p* = 0.0091. ****p < 0.0001, ***p = 0.0001, **p = 0.01, and *p = 0.05.

With an understanding of HAM cytocompatibility and cellular compartmentalization, we next sought to assess its potential prophylactic and/or therapeutic effect on oxidatively stressed cochlear cells. As oxidative stress is one of the major players in sensorineural hearing loss, these experiments are expected to serve as initial indicators of the potential prophylactic and/or therapeutic value of HAM against hearing loss. Both HEI-OC1 and SV-k1 cells were pre-treated with HAM, D-Met, or just tissue culture medium (control) and the amount of reactive oxygen species (ROS) present in each treatment group was quantified. As anticipated, stressing of the cells with H_2_O_2_ resulted in a significant increase in ROS in both cell types, with significantly higher levels detected in SV-k1 cells (Figure 3). The pre-treatment of cells with HAM yielded cellular ROS levels comparable to the unstressed controls for both HEI-OC1 and SV-k1. All the other pre-treatment conditions (D-Met or CMHA + D-Met blend) significantly reduced the amount of ROS produced by the cells, but to a significantly lesser extent than the HAM pre-treatment, and the cell line-specific differences observed in the stressed cells remained present, with SV-k1 cells displaying higher ROS levels than their HEI-OC1 counterparts. Post-stress treatment of cells with HAM, D-Met or CMHA + D-Met, did not result in any significant protective effects indicating that HAM could be potentially used as a prophylactic but not as a therapeutic (Supplementary Figure S3A). Considering that ROS are produced in the mitochondria and, as our MTS assay data indicated an increase in mitochondrial enzymatic activity in HAM-treated cells, we also sought to confirm that the observed mitochondrial enzymatic upregulation would not result in ROS production. In agreement with our assumption, our data indicated no change in ROS levels in unstressed cells, when pre-treated with HAM, D-Met or CMHA + D-Met (Supplementary Figure S3B).

### HAM Effects of Oxidative Stress-Induced Apoptosis

**FIGURE 4:**
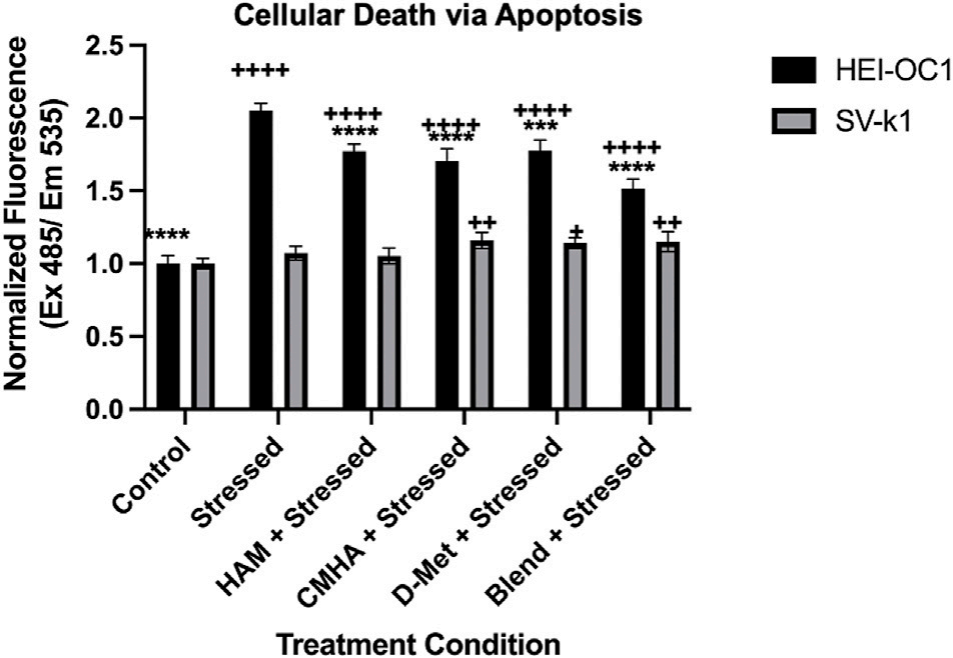
HAM effects on cellular death/apoptosis in response to oxidative stress. Data presented are mean ± SD. One-way ANOVA with Tukey’s multiple comparisons run for each cell type individually. HEI-OC1 ANOVA results compared to stressed positive control: control *p* < 0.0001, HAM *p* < 0.0001, CMHA *p* < 0.0001, D-Met *p* = 0.0001, and blend *p* < 0.0001. ****p < 0.0001 and ***p < 0.001. HEI-OC1 ANOVA results compared to control cells: stressed *p* < 0.0001, HAM *p* < 0.0001, CMHA *p* < 0.0001, D-Met *p* < 0.0001, and blend *p* < 0.0001. Indicated with ^++++^ p< 0.0001. SV-k1 ANOVA compared to stressed positive control: control *p* = 0.3932, HAM *p* = 0.9952, CMHA p0.2071, D-Met *p* = 0.4274, and blend *p* = 0.2887. ****p < 0.0001. SV-k1 ANOVA results compared to control cells: stressed *p* = 0.3932, HAM *p* = 0.6887, CMHA = 0.0042, D-Met *p* = 0.0118, and blend *p* = 0.0065. Indicated with ^++^ p< 0.01 and ^++^ p< 0.05.

As oxidative stress typically leads to cell death *via* apoptosis, we next evaluated the effect of HAM treatment on oxidatively-induced apoptosis in HEI-OC1 and SV-k1 cells *via* caspase 3/7 detection. In agreement with our ROS results, the data indicated cell type dependent levels of caspase 3 and 7 (Figure 4). Interestingly, even though SV-k1 cells expressed higher levels of ROS in response to oxidative stress, the apoptosis marker levels were lower than those in HEI-OC1. All treatment conditions appeared to reduce the amount of apoptosis markers in HEI-OC1 cells, but in SV-k1 cells, CMHA, D-Met or CMHA + D-Met blend treatments appeared to slightly increase the apoptotic marker levels. It is important to note here that overall SV-k1 cells did not appear to be particularly susceptible to oxidative stress-induced apoptosis.

### Enantiomeric and Oxidative State Effects of Methionine

**FIGURE 5:**
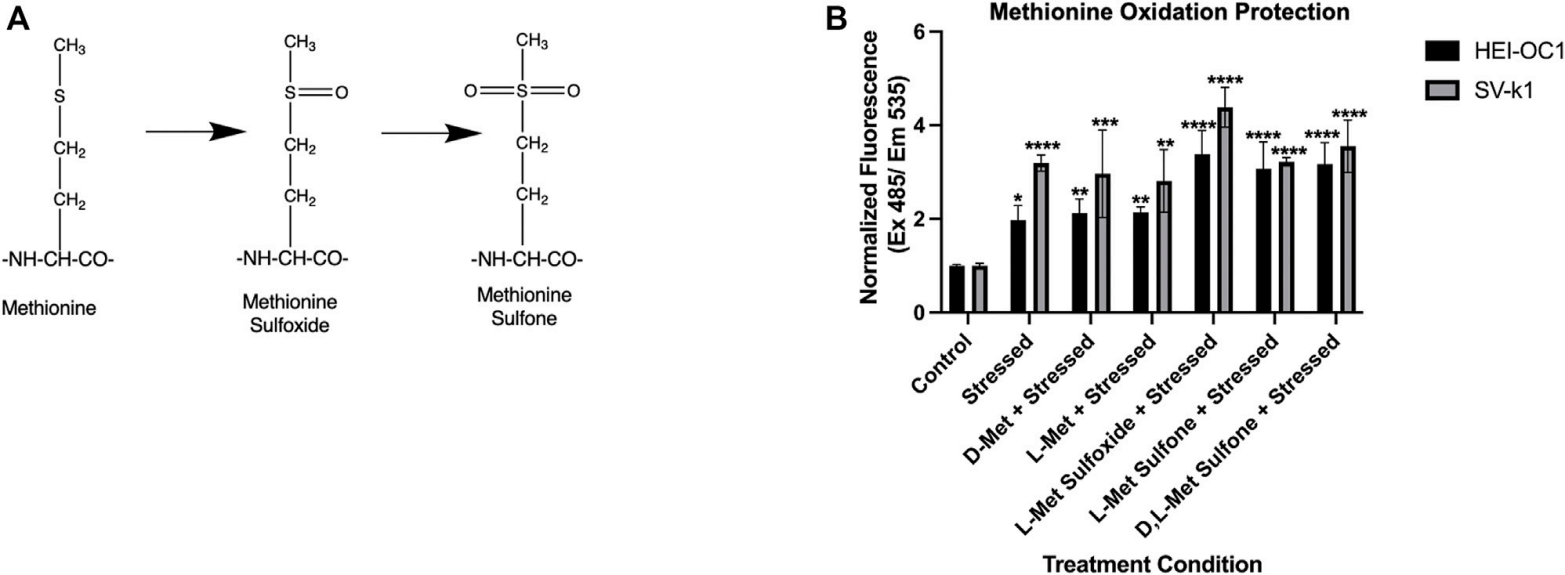
Enantiomeric and oxidative state effects of methionine. **(A)** Potential oxidative states of methionine. **(B)** Evaluation of the effects of enantiomeric and oxidative state effects of methionine on oxidatively stressed cochlear cells. Data presented are mean ± SD. One-way ANOVA with Tukey’s multiple comparisons run for each cell type individually. HEI-OC1 ANOVA versus unstressed control: stressed *p* = 0.0209, D-Met *p* = 0.0061, L-Met *p* = 0.0051, L- Met Sulfoxide *p* < 0.0001, L-Met Sulfone *p* < 0.0001, and D, L-Met Sulfone *p* = 0.0001. *p < 0.05, **p < 0.01, ****p < 0.0001. SV-k1 ANOVA versus unstressed control: stressed *p* < 0.0001, D-Met *p* = 0.0004, L-Met *p* = 0.0011, L-Met Sulfoxide *p* < 0.0001, L-Met Sulfone *p* < 0.0001, and D, L-Met Sulfone *p* < 0.0001.

Methionine has been extensively demonstrated to have antioxidant properties by directly targeting ROS and undergoing oxidation to methionine sulfoxide and methionine sulfone respectively (Figure 5A). Therefore, we sought to further investigate the insignificant D-Met protective effects observed in our ROS and apoptosis assays in both HEI-OC1 and SV-k1 cells. To assess if the enantiomeric form or oxidative status of the methionine were important, cells were treated with D-Met, L-Met, L-Met sulfoxide, L-Met sulfone and D, L-Met sulfone (Figure 5B). The results revealed several different findings. First, in agreement with the ROS data, there was no substantial reduction in the ROS levels as a result of D-Met treatment. L-Met treatment had the same effect as D-Met treatment, indicating no enantiomeric specificity in the cellular responses. Interestingly, pre-treatment of cells with methionine sulfoxide or sulfone, appeared to increase the amount of ROS detected in the cells. We hypothesize that this might be caused by the oxidative interaction of the assay reagent with the sulfoxide and sulfone. These data indicate that treatment with unconjugated D-Met or L-Met is ineffective against ROS and that methionine’s oxidative state does not have a positive impact on cellular ROS. Cumulatively, these findings appear to indicate that D-Met might not be internalized enough to trigger protective cellular effects.

### Cellular Internalization of HAM

**FIGURE 6:**
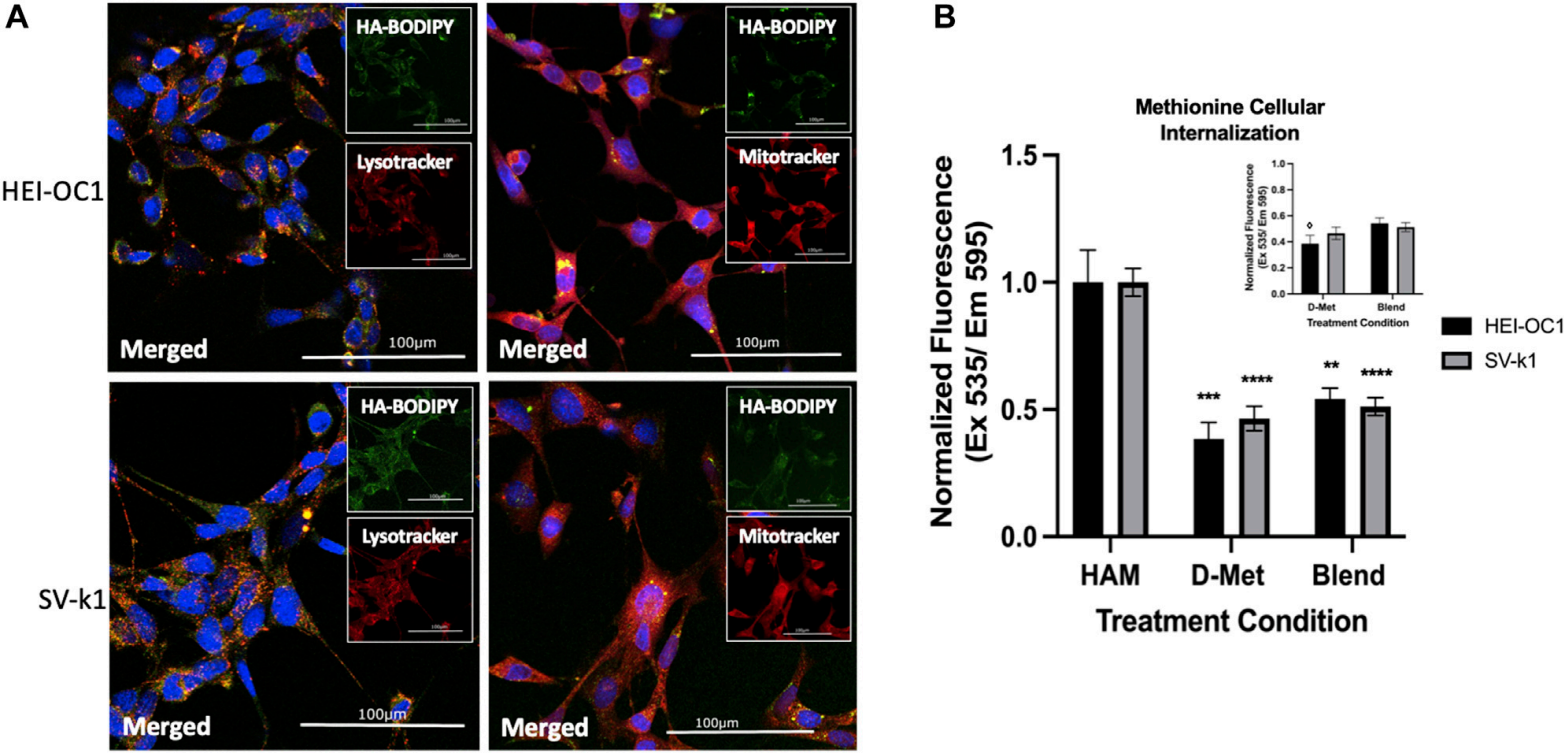
Cellular internalization of HAM. **(A)** Intracellular localization of internalized HA in HEI-OC1 and SV-k1 cells, top and bottom respectively. All scale bars 100 μm . HEI-OC1 cells with DAPI (blue), HA-BODIPY (green), and organelle tracker lysotracker on left and mitotracker on right (red). SV-k1 cells with DAPI (blue), HA-BODIPY (green), and organelle tracker lysotracker on left and mitotracker on right (red). **(B)** Quantification of the intracellular amount of D-Met. Data presented are mean ± SD. One-way ANOVA with multiple comparisons comparing cell types separately. HEI-OC1 ANOVA D-Met treated versus HAM treated *p* = 0.0003 and blend versus HAM *p* = 0.0015. SV-k1 ANOVA D-Met treated versus HAM treated *p* < 0.001 and blend treated versus HAM treated *p* < 0.0001. Inset graph is just D-Met and blend treated cells. Student t-tests indicated with ⋄ is a significant difference between D-Met and blend treated cells (*p* = 0.0238). No significant difference between SV-k1 cells treated with D-Met or blend (*p* = 0.2397).

The above-mentioned results appeared to indicate that the covalent conjugation of D-Met to CMHA was crucial for protective cellular effects, as CMHA or D-Met treatments alone or as a blend (CMHA + D-Met) did not induce similar changes in the two cochlear cell lines. HA is known to be able to internalize intracellularly *via* receptor-mediated endocytosis in various tissues. With that consideration, our assumption was that conjugation of D-Met to HA might be able to more effectively internalize the antioxidant to induce the observed changes in mitochondrial enzymatic activity and trigger cell protective effects. To test this, first, the potential internalization of unconjugated HA in the two cochlear cell lines was interrogated, *via* the use of fluorescently labeled-HA (HA-BODIPY) and microscopical visualization of the compound’s cellular distribution. Our data indicate that in both cell types, HA was able to relocate inside the cells and appeared to co-localize with lysosomes (stained with LysoTracker) and mitochondria (stained with MitoTracker), although its exact organelle-specific location could not be pinpointed at this resolution (Figure 6A). In the context of these observations, however, a mitochondrial localization would be in line with our observed MTS assay results.

Second, we sought to assess the amount of D-Met internalized when cells were treated with conjugated versus unconjugated antioxidant. For this HEI-OC1 and SV-k1 cells were treated with HAM, D-Met or CMHA + D-Met and the content of intracellular methionine was quantified. The obtained data indicate that treatment of cells with D-Met alone or as blend with CMHA resulted in significantly less intracellular D-Met than in the cell lysates treated with HAM (Figure 6B). Interestingly, the data also seem to indicate that in the HEI-OC1 cells the blend of D-Met and CMHA did increase the intracellular concentration of D-Met compared to the D-Met alone treatment (Figure 6B inset). Nevertheless, these changes were insignificant compared to the effects of HAM treatment on the amount of intracellular D-Met. Overall, these results further highlight the impact of the chemical conjugation between D-Met to CMHA in the context of the development of a novel compound targeting hearing loss.

### Potential Protective Mechanisms of HAM

**FIGURE 7:**
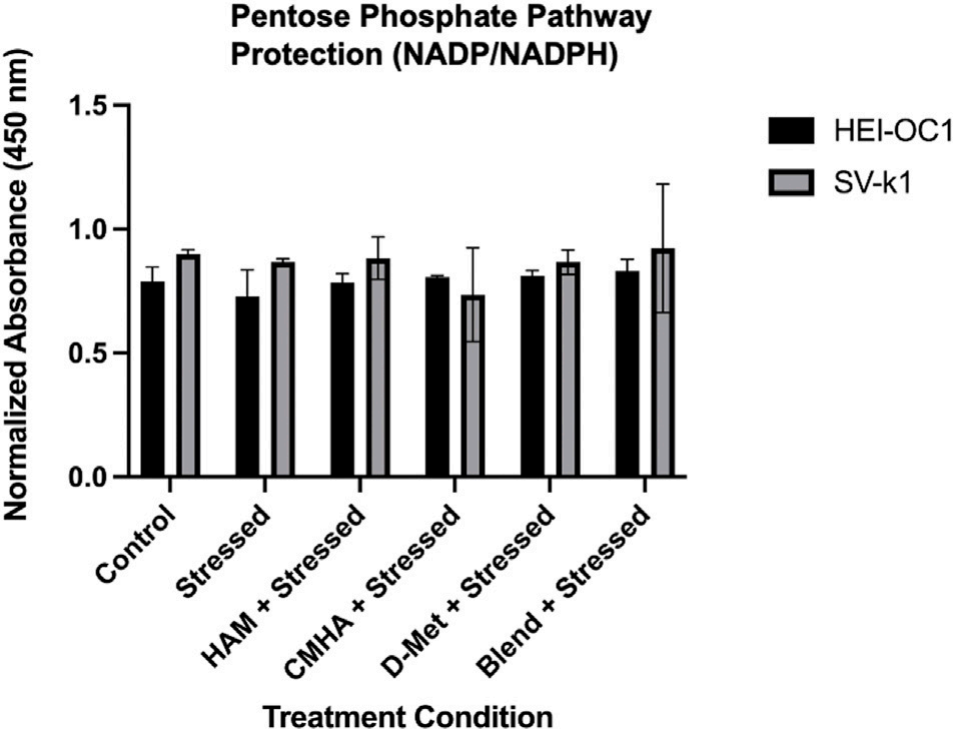
Investigation of the potential mechanism of HAM protection. Data presented are mean ± SD. One-way ANOVA with Tukey’s multiple comparisons run for each cell type individually. HEI-OC1 ANOVA versus unstressed control: stressed *p* = 0.7694, HAM *p* > 0.9999, CMHA *p* = 0.9989, D-Met *p* = 0.9964, and blend *p* = 0.9391. SV-k1 ANOVA versus unstressed control: stressed *p* = 0.9996, HAM *p* > 0.9999, CMHA *p* = 0.6927, D-Met *p* = 0.9997, and blend *p* > 0.9999.

With the D-Met cellular internalization confirmed, the next step was to interrogate the potential mechanism of protection employed by the internalized antioxidant. One such mechanism could involve the modulation of the NADP/NADPH ratio in the pentose phosphate pathway (PPP). To investigate this potential mechanism, HEI-OC1 and SV-k1 cells were treated with HAM, D-Met or CMHA + D-Met and the NADP/NADPH ratio was quantified. Both cell lines showed no significant difference in the NADP/NADPH ratio in any of the treatment groups or control groups (Figure 7). These results suggest that mechanisms other than upregulation of the NADP/NADPH ratio in the PPP pathway are responsible for the observed protective effects; however, further mechanistic investigations were out of the scope of this study.

### HAM Permeation Through an Anatomical Barrier

The original intent of this study was to develop a topically applicable prophylactic or therapeutic against hearing loss. Therefore, the next step in HAM’s characterization process was to assess its ability to permeate through biological membranes typical for ear structures. Specifically, a therapeutic targeting the inner ear would be expected to permeate at a minimum the round window membrane (RWM). To test this, we used a three-dimensional tissue model of RWM, consisting of underdeveloped airway cells.

**FIGURE 8:**
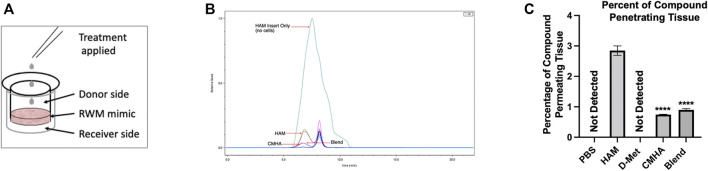
Investigation of the transmembrane permeation ability of HAM. **(A)** Illustration of the permeation experiment setup. **(B)** Chromatogram of SEC analyzed samples detected with an RI detector. The teal (largest) curve corresponds to HAM passed through a blank/no tissue insert. The mustard green and purple traces correspond to HAM passed through inserts with Epi-Airway tissues as round window membrane (RWM) models. The magenta trace corresponds to the blend treatment passed through an insert with Epi-Airway, and the dark purple trace corresponds to CMHA passed through an insert with Epi-Airway. **(C)** Quantification of the amount of HAM or controls detected after permeation of Epi-Airway tissues. Data presented are mean ± SD. One-way ANOVA with Tukey’s multiple comparisons run. ANOVA results compared to HAM group: CMHA *p* < 0.0001 and blend *p* < 0.0001.

Therefore, HAM and controls were then placed on top of the tissues (donor side) and the amount that traveled through the tissue (receiver side) was quantified (Figure 8A). The receiver side solutions were then analyzed with two SEC columns: one for higher molecular weights (6 kDa–10 MDa) capable of detecting HAM, and CMHA and one for lower molecular weights (100 Da–20 kDa) capable of detecting free D-Met (Figure 8B). Our data indicate that HAM and CMHA were both able to permeate the RWM model, at 2.93% and 0.77%, respectively, of the donor side-applied amount (Figure 8C). Interestingly, the CMHA amount that permeated the model when applied to the donor side as blend, was slightly higher, at 0.90%, than when applied individually. For D-Met detection a standard curve based on antioxidant-specific absorbance A235 values were created to ensure proper chromatographic detection and quantification of the antioxidant (Supplementary Figure S4). However, all treatment conditions resulted in no detectable levels of D-Met in the receiver solution. These results could indicate that the amount of antioxidant able to penetrate the RWM model was either insignificant or below the detection threshold of our instrument (0.01 mg/ml) in the D-Met only or CMHA + D-Met treated samples, and that D-Met was most likely still covalently attached to the CMHA backbone in the HAM treated sample (Supplementary Figure S5). To further assess the latter, we used absorbance A235 detection on the large molecular weight range SEC column and quantified the area under the curve (AUC) for HAM and CMHA, before and after permeation through the tissue models. The analyses indicated a significantly higher AUC of 958.419 for HAM compared to the CMHA only treated group, that had an AUC of 12.945. These values were similar to those obtained for control HAM and CMHA that have not been permeated through the tissue models (Supplementary Table S3). Overall, our data indicate that a significantly higher amount of HAM permeated the tissue compared to CMHA only or CMHA + D-Met blend, and it appears that D-Met remains covalently attached to the CMHA backbone throughout the RWM model permeation.

## Discussion

The goal of this study was to assess the feasibility of developing a topically applicable prophylactic or therapeutic against hearing loss. As oxidative stress is one of the major factors associated with hearing loss, regardless of its pathogenesis (Kamogashira et al., 2015; Kurabi et al., 2017), our approach was to use a well characterized antioxidant (D-Met) (Tavanai and Mohammadkhani, 2017; Pecha et al., 2020) and chemically attach it to a “carrier” to facilitate the permeation of ear specific biological barriers such as the round window membrane and delivery to the sensory cells of the cochlea. As a carrier we selected HA, as previous literature reports indicate that drug blends with HA had increased permeation rates across the tympanic and/or round window membrane (Shibata et al., 2012; Queisser et al., 2021). Additionally, the polymeric structure of HA and the presence of readily modifiable chemical functionalities translate this macromolecule into an excellent building block for more complex structures.

**FIGURE 9:**
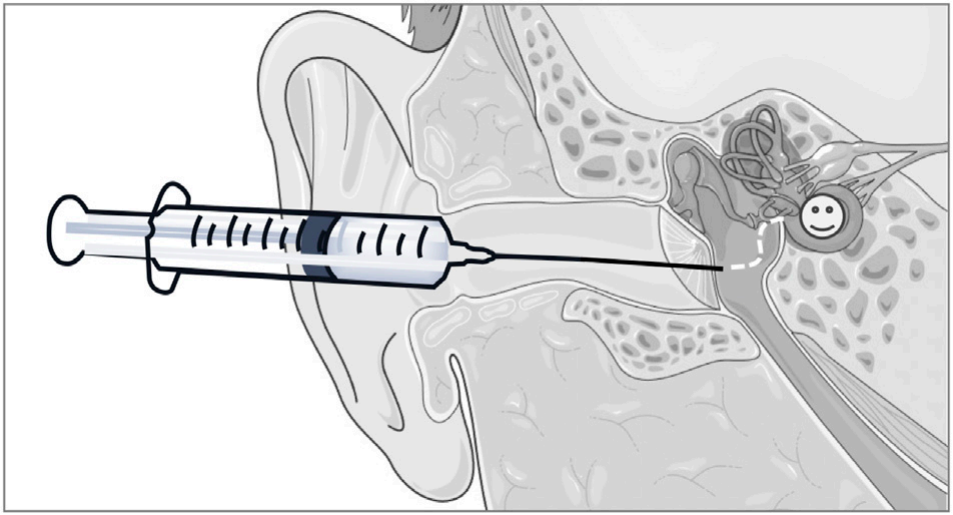
Illustration of the rationale for the development of a liquid formulation that could be topically deployed into the middle ear of patients, from where it could subsequently, permeate across the RWM to access the cochlear structures susceptible to oxidative damage. The schematic art pieces used in this figure were created by using Servier Medical Art (http://servier.com/Powerpoint-image-bank). Servier Medical Art by Servier is licensed under a Creative Commons Attribution 3.0 Unported License.

From a practical, clinically applicable product perspective, our rationale was to develop a liquid formulation that could be topically deployed into the middle ear of patients (i.e., *via* intratympanic injections), from where it could permeate across the RWM to access the cochlear structures susceptible to oxidative damage (Figure 9). As a first step in our approach, the HA backbone was enriched in carboxyl functionalities to obtain CMHA, which contained additional functionalization sites (58% increase in -COOH groups) (Serban and Kaplan, 2010; Love et al., 2019). Next, we chose a well characterized carbodiimide mediated crosslinking strategy to covalently connect -COOH (from the CMHA backbone) and -NH_2_ groups (from the antioxidant) (Serban and Prestwich, 2008; Serban and Skardal, 2019) and showed that we can tailor the amount of covalently conjugated D-Met simply by changing the reaction stoichiometry. As the 10X and 20X stoichiometries generated comparable degrees of CMHA functionalization, the 10X reaction product was further characterized as it was expected to reflect the same biological effects as the 20X version but with lower cost of production. For cellular effects evaluations of two different mouse cochlear cell lines were used: HEI-OC1, derived from the organ of Corti, and SV-k1, derived from the stria vacularis, as both cell types were shown to be responsible for normal hearing (Liu et al., 2016). HEI-OC1 cells are organ of Corti-derived and have been used to investigate cellular events such as drug-activated apoptotic pathways, senescence, mechanism of cell protection, inflammatory responses, effects of hypoxia, oxidative and endoplasmic reticulum stress, and expression of molecular channels and receptors (Kalinec et al., 2003; Kalinec et al., 2016). SV-k1 cells are derived from the Immortomouse^™^ stria vascularis and have been used as models for strial capillary basement membrane studies (Gratton et al., 2002; Rivolta and Holley, 2002; Kalinec et al., 2016). Cytocompatibility evaluation assays revealed that HAM was well-tolerated by both types of cochlear cells; however, interestingly, in both cell lines HAM appeared to upregulate the activity of mitochondrial enzymes, although to a significantly higher extent in the SV-k1 cells. When evaluated for oxidative protection effects in response to hydrogen peroxide-induced oxidative stress, HAM pretreatment of the cells kept ROS levels similar to those in the control/unstressed cells. Interestingly, our data revealed that the two cell lines have different susceptibility to oxidative stress, with SV-k1 cells generating a significantly higher amount of ROS in response to oxidative stress, compared to HEI-OC1 cells. Additionally, treatment of cochlear cells with HAM after oxidative stress did not show a decrease in ROS levels, suggesting that HAM could be used as a prophylactic but not as therapeutic.

Oxidative stress is typically associated with apoptosis (Huang et al., 2000), therefore our expectation was that the two cochlear cell lines used would undergo apoptosis in response to hydrogen peroxide treatment. We chose to monitor this by the assessment of caspase 3/7 levels in response to oxidative stress. Caspase 3 and caspase 7 are considered universal apoptosis markers that are both activated regardless of the specific cell death inducing stimulus (Walsh et al., 2008). Surprisingly, the levels of apoptotic markers were similar to the unstressed controls in SV-k1 cells while in HEI-OC1 cells the apoptotic marker levels were significantly higher than in unstressed cells. However, HAM pre-treatment of the cells significantly reduced the amount of Caspase 3/7 in HEI-OC1 cells, although they were still much higher than in the unstressed controls. All the aforementioned biological effects were specific to HAM and were not observed with CMHA, D-Met and CMHA + D-Met blend pre-treated samples, highlighting the importance of the covalent attachment of D-Met to the CMHA backbone for protective effects initiation.

In most tissue types, HA is cellularly internalized *via* HA-specific receptor mediated processes (Misra et al., 2015). The main HA receptor responsible for internalization is Cluster of Differentiation 44 (CD44); however, currently there are very limited data available on the presence of CD44 or endogenous HA in cochlear structures (Vilstrup and Jensen, 1954; Friberg et al., 1989; Anniko and Arnold, 1995; Hertzano et al., 2010). A survey of the gEAR multi-omic database (Orvis et al., 2021) indicated that CD44 is expressed in murine cochlear structures at the mRNA level (Hertzano et al., 2010). Therefore, based on our previous findings that HAM pre-treatment increased cochlear cell mitochondrial enzymatic activity, and that chemical conjugation was needed for protective effects, we postulated that HAM was internalizing D-Met to trigger oxidative protection responses. We tested this hypothesis through two different approaches. First, we showed that, at the concentrations tested, D-Met by itself was not able to enter cochlear cells at relevant levels and this effect was independent of its enantiomeric specificity or oxidation status. Second, we showed that HAM did facilitate the internalization of D-Met and that this is most likely due to the natural propensity of (CM)HA to be internalized *via* HA-specific receptors. Our data (MTS assay and HA-BODIPY cellular tracking) indicate that HA and HAM are co-localizing with the mitochondria and potentially with mitochondrial-lysosomal complexes (Todkar et al., 2017). In other tissues, HA has been reported to localize in the lysosome (Xue et al., 2020); therefore, further investigation of the exact location of HAM in cellular structures would be warranted, especially given that the amount of information available on the role of HA in cochlear structures and hearing is extremely scarce. We were unable to pinpoint the exact mechanism of protection, although we excluded the involvement of the PPP pathway, or at least the involvement of the PPP-associated NADP/NADPH ratio (Kuehne et al., 2015). Collectively, our data revealed that HAM has promising prophylactic and protective properties if applied to cochlear cells prior to oxidative stress.

Circling back to our original study goal, the remaining step was to test the ability of HAM to permeate ear-specific biological membranes unaltered, specifically without “loosing” the attached antioxidant. A previous study (Liebesny et al., 2018) suggested that airway cells could be a viable model for the RWM. Therefore, we used three-dimensional underdeveloped (prior to development of cilia) airway tissues similar in thickness to the physiological RWM, to assess HAM’s ability to effectively carry across its antioxidant cargo. Our data suggest that not only is HAM able to efficiently permeate the RWM model at higher rates than CMHA or D-Met only, or CMHA + D-Met blend respectively, but D-Met remains covalently attached throughout the process. Cumulatively, our data seem to indicate that if HAM would be delivered into the middle ear space (i.e., *via* intratympanic injections), it would be able to then carry its antioxidant cargo effectively across the RWM as conjugated HAM, and protect cochlear cells from oxidative damage *via* D-Met internalization as HAM and subsequent intracellular D-Met release, as indicated by our HEI-OC1 and SV-k1 data.

In summary, we have successfully synthesized and characterized a novel HA derivative that shows promise as a prophylactic against oxidatively induced hearing loss, in addition to being able to carry the attached antioxidant cargo across ear-specific anatomical barriers. Further *in vivo* studies in animals with age, noise or drug-induced hearing loss will be needed to confirm our findings, and the overall role of HA in the cochlea would further need to be investigated; however, we believe that we have successfully shown that covalent conjugation of a therapeutic molecule to an HA carrier is a viable approach to subsequent prophylactics and therapeutics development against hearing loss.

## Data Availability

The raw data supporting the conclusion of this article will be made available by the authors, without undue reservation.
