# Supplementary material for: Development and Characterization of a Topically Deliverable Prophylactic Against Oxidative Damage in Cochlear Cells
Source: Front Pharmacol. 2022 Jun 9;13:907516. doi: 10.3389/fphar.2022.907516 (PMC9226984; doi:10.3389/fphar.2022.907516)
Supplement: Supplementary file 1 [file DataSheet1.docx]

Supplemental Material

**Methods**

**Reactive Oxygen Species (ROS) Detection Post-Stress Treatment**

HEI-OC1 and SV-k1 cells, respectively, were seeded in a 96-well plate at a seeding density of 2 x 10^5^ cells/mL in DMEM media with 10% FBS and incubated at 33℃/10% CO2. The cells were allowed to adhere 24 hours, then the media was aspirated and replaced with 1 µM CM-H2DCFDA in PBS and incubated for 30 minutes. The reagent was then aspirated and replaced with either PBS for control or 0.5 mM H_2_O_2_ in PBS and incubated for 30 minutes. Subsequently, the media was aspirated, cells were washed twice with PBS followed by the addition of sterile filtered media containing HAM conjugates and controls, and incubation for 24 hours. Media was then aspirated, and wells were washed 2 times with PBS. The amount of ROS was quantified using florescence (ex/em 495/530 nm) with the FilterMax F5 multimode microplate reader.

**Amino acid analyses**

Amino acid composition was analyzed per a previously published protocol ^1^(Numata and Baker, 2014). Briefly, the powder sample was hydrolyzed with 6 M HCl at 110 °C for 20 hours in an evacuated sealed tube. The hydrolysate was sonicated in 100 µL of 0.25 M 0.25 M lithium citrate buffer (pH 2.2) for 15 minutes and then filtered. The filtrate was diluted 10 times in 0.25 M lithium citrate buffer (pH 2.2). Diluted samples (50 µL volumes) were then injected onto High-Speed Amino Acid Analyzer L-8900. Free amino acids in the sample were separated by ion exchange and derivatized with ninhydrin for detection at 570 or 440 nm. The natural amino acids and D-Met were used to calibrate the amino acid analyzer.


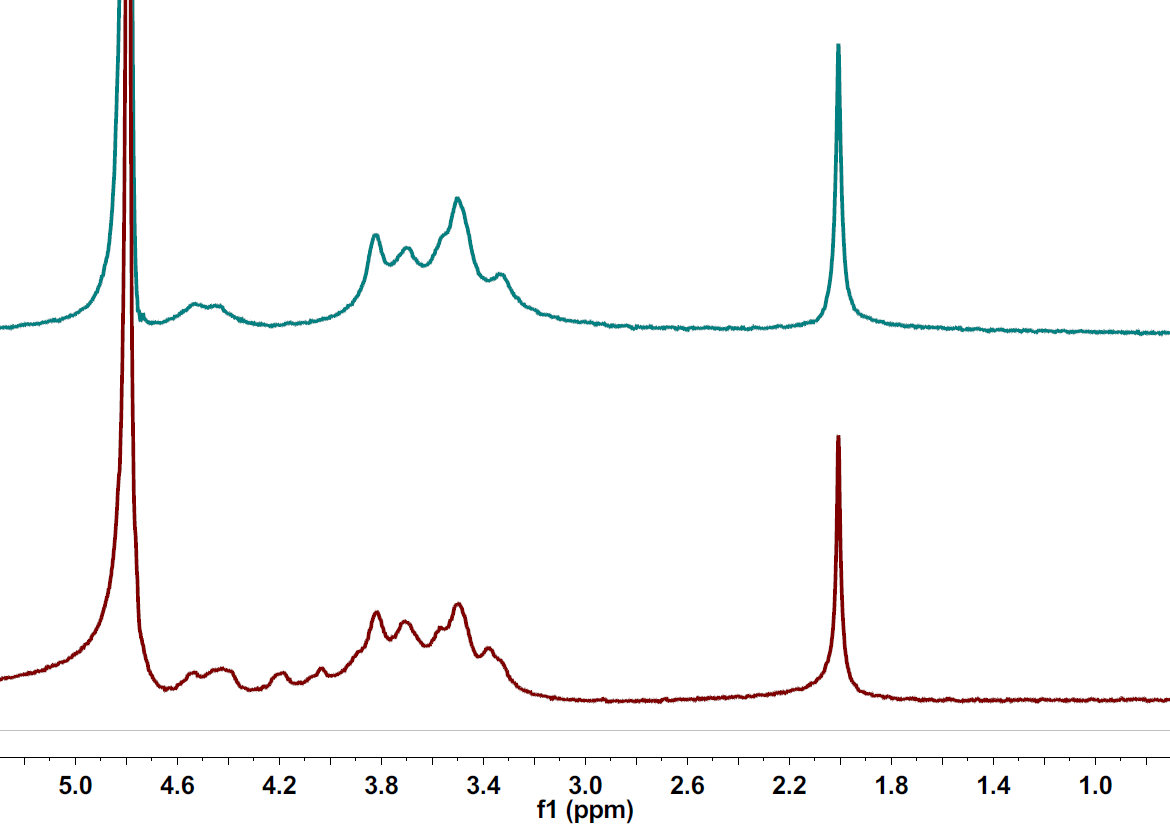


**HA**

**CMHA**

A


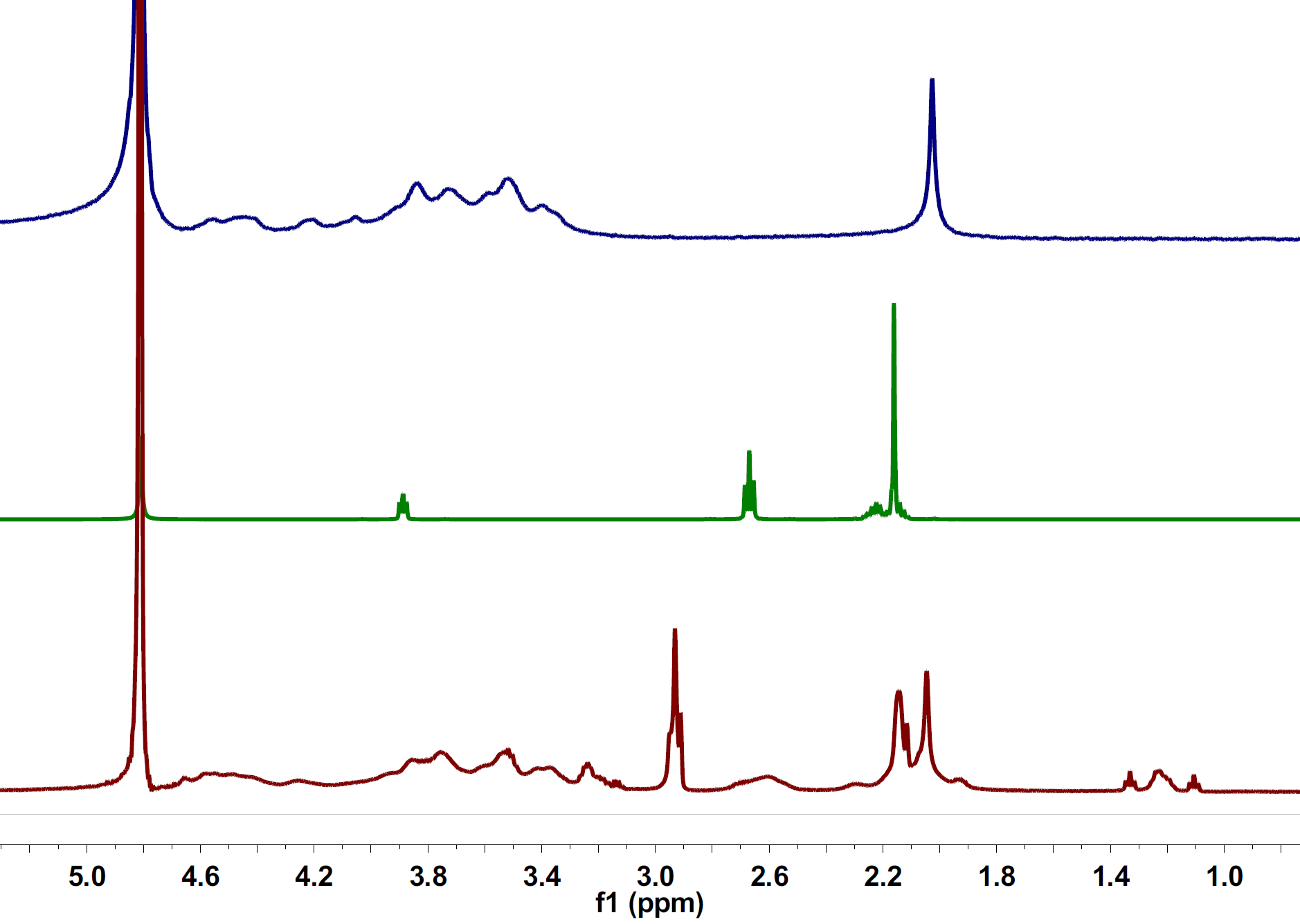


**CMHA**

**D-Methionine**

**HAM**

B

**Supplemental Figure 1**. A) ^1^H-NMR of hyaluronic acid (HA) and carboxymethylated hyaluronic acid (CMHA). Grey highlight shows area of interest. B) H-NMR of carboxymethylated hyaluronic acid (CMHA), D-Methionine and D-Methionine conjugated to carboxymethylated hyaluronic acid (HAM). Grey highlight shows area of interest.


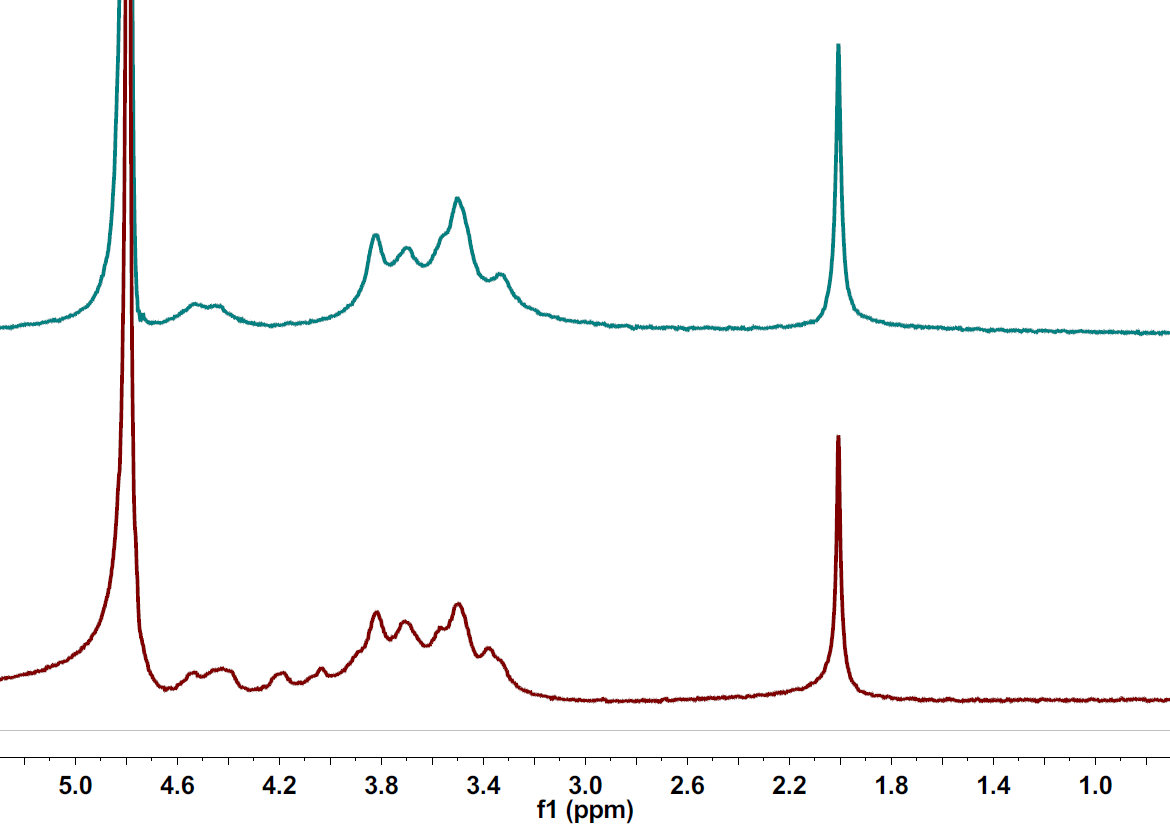


**HA**

**CMHA**

$$\% carboxylation= \frac{CH_{2}COOH}{2} x 100$$

$$58 \% carboxylation= \frac{1.16}{2} x 100$$

**Supplemental Figure 2**. Quantification of carboxymethylation. ^1^H-NMR of hyaluronic acid (HA) and carboxymethylated hyaluronic acid (CMHA). Grey highlights show peaks integrated. Methyl protons originating from the N-acetyl glucosamine unit (δ=2.01 ppm) set to 3. Then the integration signal from methylene protons of attached carboxymethyl groups (δ=4.19 ppm) were divided by two and multiplied by 100 to determine percent of carboxymethylation.


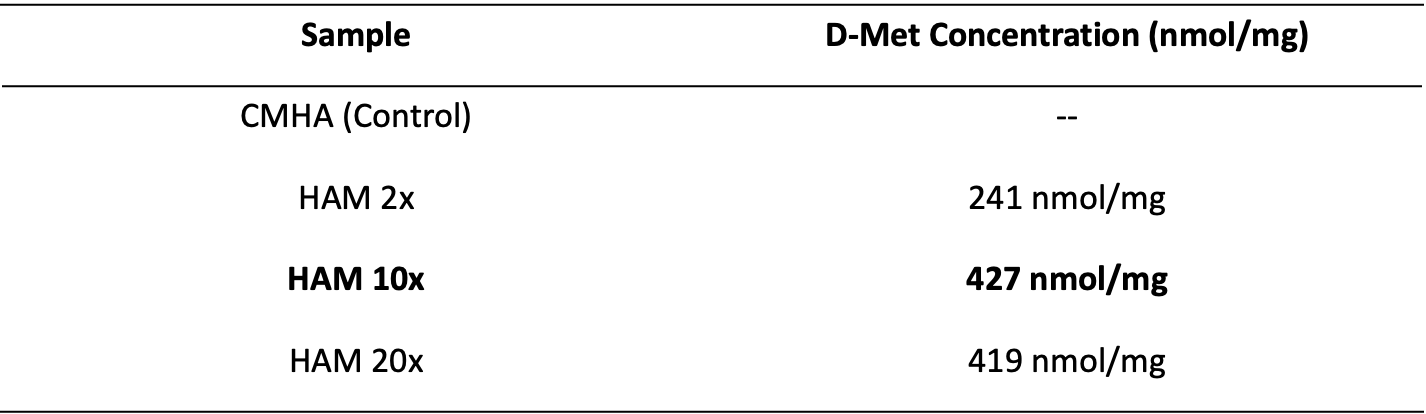


**Supplemental Table 1.** Amino acid analyses of HAM conjugates obtained with different reaction stoichiometries. The 10X conjugate (bold font) was chosen for further characterization.


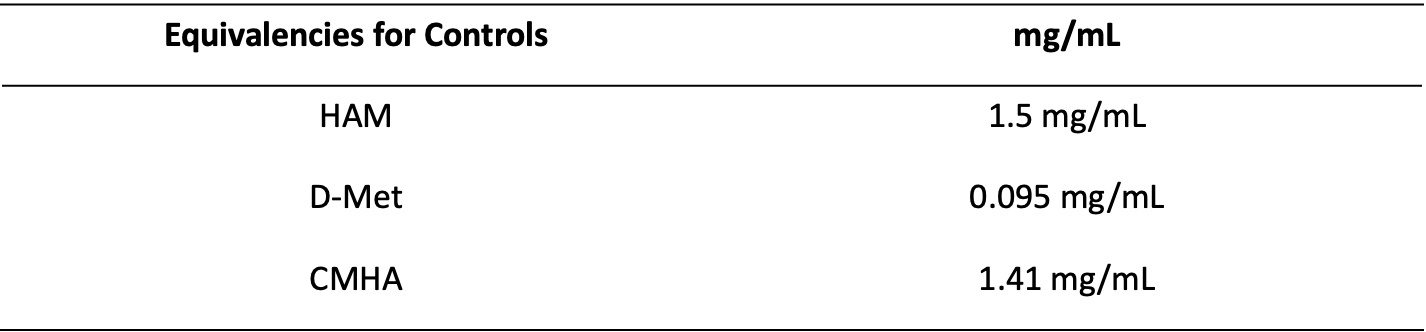


**Supplemental Table 2.** HAM equivalent amounts of CMHA and D-Met used as controls either alone or as blend in subsequent experiments.

A

B

**
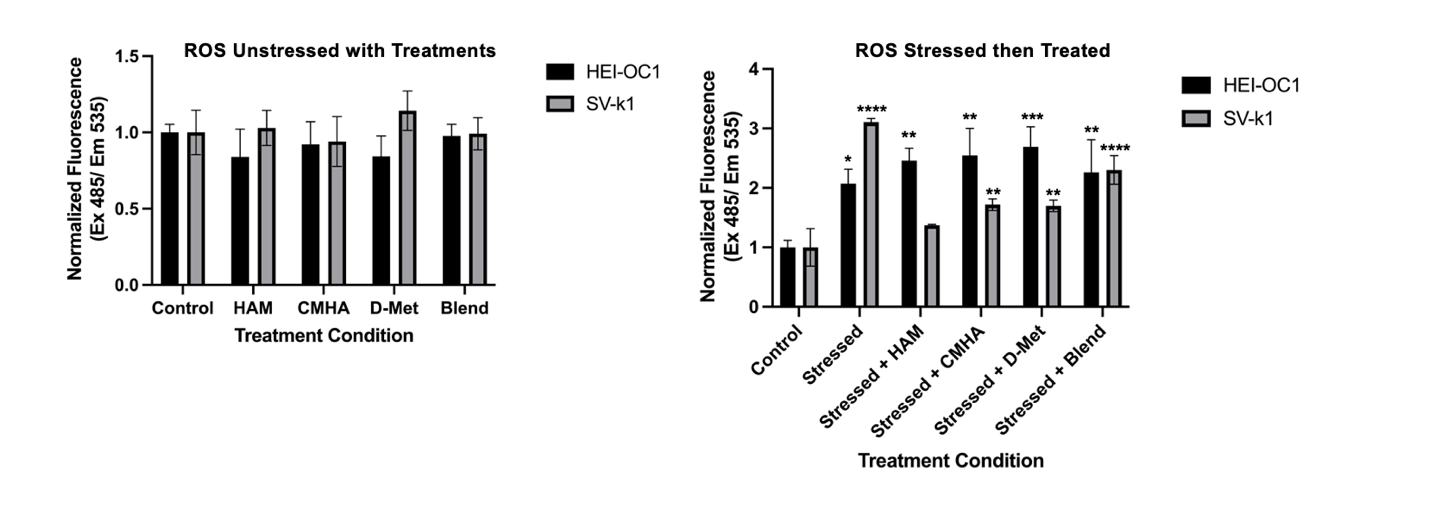
**

**Supplemental Figure 3. A**) Reactive oxygen quantification in control cells (not stressed with 0.5 mM hydrogen peroxide) after treatment with HAM and controls. Data presented is mean ± SD. One-way ANOVA with Tukey’s multiple comparisons run for each cell type individually. HEI-OC1 ANOVA results compared to unstressed control: HAM p=0.5459, CMHA p=0.9598, D-Met p=0.5741, and blend p=0.9998. **** p<0.0001. SV-k1 ANOVA results compared to unstressed control: HAM p=0.9995, CMHA p=0.9866, D-Met p=0.6559, and blend p>0.9999. ****p<0.0001. **B**) Reactive oxygen quantification in cells stressed with 0.5 mM hydrogen peroxide, and subsequently treated with HAM and controls after being stressed with 0.5 mM hydrogen peroxide. HEI-OC1 ANOVA results compared to unstressed control: stressed p=0.0270, HAM p=0.0029, CMHA p=0.0018, D-Met p=0.0008, and blend p=0.0088. SV-k1 ANOVA results compared to unstressed control: stressed p<0.0001, HAM p=0.1663, CMHA p=0.0030, D-Met p=0.0037, and blend p<0.0001.

**
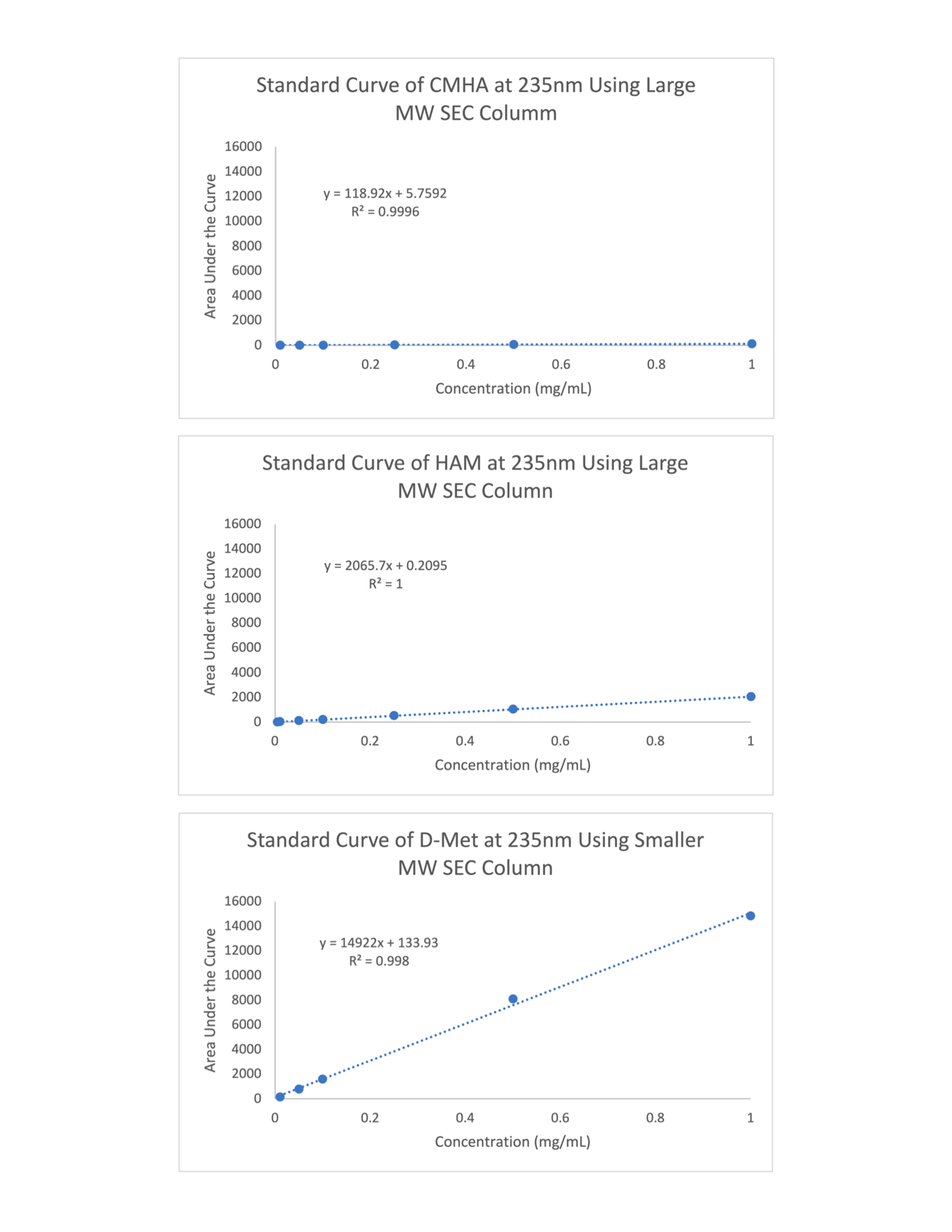
Supplemental Figure 4.** Standard curves of HAM, CMHA and D-Met AUC via HPLC UV-Vis detector at 235 nm.


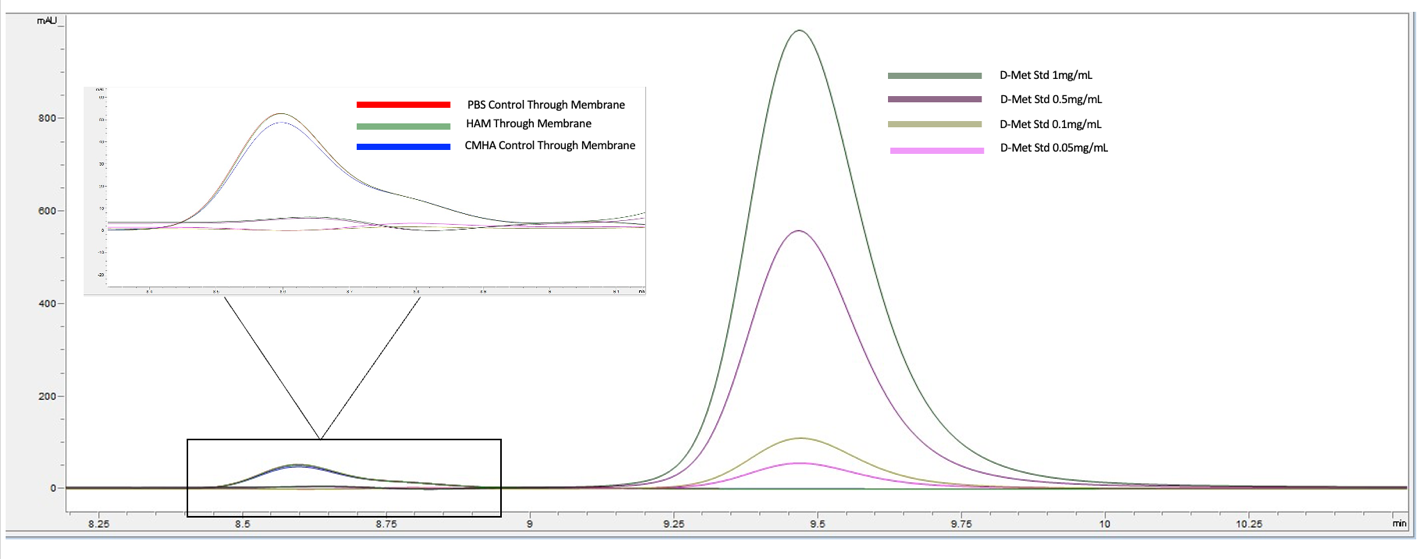
**Supplemental Figure 5.** SEC chromatogram using the column for smaller molecular weights. Shows D-Met standard curve while inset shows zoomed in peaks for PBS control, HAM, and CMHA after permeation experiment.

| **Treatment Conditions** | **Averaged AUC at 235 nm (n = 3)** |
| --- | --- |
| HAM Through Tissue | 958.419 |
| CMHA Through Tissue | 12.945 |
| Blend Through Tissue | 63.323 |
| HAM no Tissue (0.05mg/mL) | 204.145 |
| CMHA no Tissue (0.05mg/mL) | 22.762 |

**Supplemental Table 3.** Averaged AUC at 235 nm of HAM, CMHA and blend after permeation experiment and prior to permeation experiment.

Numata, K.; Baker, P. J. Synthesis of Adhesive Peptides Similar to Those Found in Blue Mussel (Mytilus Edulis) Using Papain and Tyrosinase. *Biomacromolecules* **2014**, *15* (8), 3206–3212. https://doi.org/10.1021/bm5009052.
